# Editorial: Dietary habits, microbiota and autoimmune diseases

**DOI:** 10.3389/fnut.2023.1233863

**Published:** 2023-06-23

**Authors:** Manuela Berto Pucca, Julio Villena, Gislane Lelis Vilela de Oliveira

**Affiliations:** ^1^Department of Clinical Analysis, School of Pharmaceutical Sciences, São Paulo State University (UNESP), Araraquara, Brazil; ^2^Laboratory of Immunobiotechnology, Reference Centre for Lactobacilli (CERELA-National Council of Scientific and Technological Research), San Miguel de Tucumán, Argentina; ^3^Food and Feed Immunology Group, Laboratory of Animal Products Chemistry, Graduate School of Agricultural Science, Tohoku University, Sendai, Japan; ^4^Microbiology Program, Department of Food Science and Technology, Institute of Biosciences, Humanities and Exact Sciences (IBILCE), São Paulo State University (UNESP), São José do Rio Preto, Brazil; ^5^Laboratory of Immunomodulation and Microbiota, Department of Chemical and Biological Sciences, Institute of Biosciences (IBB), São Paulo State University (UNESP), Botucatu, Brazil

**Keywords:** nutrition, microbiota-immune system axis, gut microbiota, immune-mediated diseases, autoimmune diseases

Particular emphasis is given to the dietary habits as one of the modulators of the microbiota-immune system axis, pointing to the importance of studies in the interface of nutrition, microbiology and immunology (1, 2). Studies showed that macronutrients, micronutrients, and the different dietary profiles shape the intestinal microbiota diversity and their metabolities, impacting the human health or disease (3–7). It is already known that the microbiota establishment in early-life plays an essential role in the development and maturation of mucosal and systemic immune system (8–11). In addition, the complex interaction between available nutrients and the intestinal microbiota are able to impact our immune responses throughout life, maintaining homeostatic conditions (12–15). The breakdown of this balanced diet-microbiota-immune system crosstalk can influence disease triggering, including immune-mediated diseases, including autoimmune and allergic diseases (16–18).

The autoimmune diseases are multifactorial conditions, involving genetic and environmental factors, as well as, a dysfunctional immune response and intestinal dysbiosis (19–21). Besides that, the increased incidence of autoimmune diseases worldwide (3–9%) is not explained by genetic background or infections, suggesting a fundamental role of westernized dietary habits and dysbiosis in disease development in industrialized societies (22–26). The nutrition-mediated disruption of the microbiota-immune system axis can lead to short and long-term effects, such as dysbiosis, mucosal barrier deterioration, leaky gut, microbial translocation to lamina propria and bloodstream, and systemic inflammation, impacting all of the gut-organ axis and our entire physiology (20, 27–30). Westernized diet includes high consumption of saturated fats, sugar, additives, and decreased fibers' intake (31, 32). Mediterranean or plant-based diets include high consumption of fruits, vegetables, whole-grains, including fibers and high quality fats (33–36). Western and Mediterranean diets are representative models of detrimental or beneficial dietary patterns, respectively (31).

The future of the microbiota-based nutrition is to prevent diseases in genetic predisposal individuals, and predict clinical phenotypes and diseases in order to guide personalized therapies. Moreover, the personalized microbiota-based diets could be designed using the computational machine learning to design diets that will modulate the microbiota-immune system axis and improve clinical responses in autoimmune diseases (37–42).

In this Research Topic of Frontiers in Nutrition, we aimed to assemble a series of articles that highlight how the dietary habits impact the microbiota-immune system axis, and could influence immune-mediated diseases. We aimed to collect studies that report mechanisms and personalized dietary-based interventions to modulate the microbiota-immune system axis and immune-mediated diseases. A total of six articles were accepted and 28 researchers participated in this Research Topic.

First of all, in a perspective article, Larsen discussed the possible factors involved in rising incidence of autoimmune diseases and the “old friends hypothesis” that states that “the decreased exposure to microbes/infections early in life promote a defective immune system maturation” and could be involved in worldwide spread of immune-mediated diseases. Also, the modulation of the gut microbiota though dietary interventions in young age could represent a preventive, as well as a therapeutic strategy in autoimmune diseases.

Multiple sclerosis (MS) is an autoimmune, chronic, inflammatory neurodegenerative disease of the central nervous system that affects young adults, and twice women (43). It is estimated that 2.8 million subjects are currently diagnosed with the disease, and the worldwide prevalence increased 30% since 2013, and are partially attributed to environmental changes, including industrialization and westernization diets, alterations of the gut microbiota, and intestinal permeability (44). The intestinal dysbiosis, leaky gut and bacterial translocation have been associated with MS susceptibility and progression (45). Hoffman et al. revised studies showing dysbiosis in mice with experimental autoimmune encephalomyelitis (EAE), and in MS patients. Then, authors compiled works concerning the influence of dietary components on EAE and MS patients, including the beneficial effect of dietary fibers, their metabolites and polyunsaturated fatty acids on neuroinflammation, as well as, supplementation with A, D, and E vitamins. Besides that, the mini-review discussed some dietary interventions applied in EAE models and in clinical settings, including Mediterranean and isoflavone diets, plant-based/low-protein intake, ketogenic diet, and intermittent fasting. Finally, researchers concluded that microbiota-based therapies through dietary interventions could function as adjunctive strategy for MS treatment.

Similarly, Bronzini et al. discussed how food-microbiota axis could support intestinal dysbiosis, immunologic deregulation and neuroinflammation in MS, favoring disease onset and progression. In addition, researchers included preclinical reports evaluating the role played by high-fat, high-salt, high-sugar diets on gut microbiota of EAE mice, as well as, the influence of dietary fibers, tryptofan, isoflavones, and calorie restriction in the gut-immunity on EAE. In MS patients, studies involving the impact of Mediterranean, ketogenic, low-salt, and calorie restriction diets were also considered as dietary intervention. To finalize the review, the authors discussed the applications of prebiotics, probiotics, and post-biotics as adjuvant therapy to treat MS, considering their capacity to induce an anti-inflammatory milieu and to provide metabolites that cross-feeds beneficial microbes from the gut microbiota.

The inflammatory bowel diseases (IBD) include chronic inflammatory conditions of the gastrointestinal tract, including Crohn's disease and ulcerative colitis. The worldwide IBD prevalence increases and affects 1 in 200 young subjects in Western countries (46). Crohn's disease (CD) involves genetic and environmental factors in the disease development, including deregulated immune responses against the commensal gut microbiota, and is characterized by chronic and progressive inflammation of the gastrointestinal tract (47, 48). Ulcerative colitis (UC) is a chronic inflammatory bowel disease that affects the colon and the rectum (49). Deficiencies in micronutrients' absorption are normally observed in patients with IBD (50, 51). The review from Wu et al. summarized the role of vitamins and minerals' supplementation in IBD patients, as well as the importance of monitoring these patients and replacement therapy for these nutrients.

The last two original articles discussed important aspects of the preference for ethanol and its relationship with immune and the central nervous systems and the association between dietary factors and asthma. It was proposed that in the brain integrating region of reward system, the striatum, the interaction of the immune response and the expression of the Lrrk2 gene is involved in the addiction to ethanol (52). In the article of Moreira-Júnior et al., the preference for ethanol and its relationship with the interplay between the immune and the central nervous systems was investigated. By using wild-type C57BL/6 mice as well as Il6-/- and Nfat-/- animals, the authors tested whether high fat and sugar diet intake and free choice for ethanol altered the expression of Lrrk2 and immune genes. The work reported an increased compulsive-like behavior, an up-regulation of inflammatory genes (Il6, Il1β, iNOS Tlr4, and Nfat) as well as a down-regulation of the regulatory cytokine Il10, the Lrrk2 gene, and the dopamine receptor (Drd2) in the striatum. It was also observed that the absence of Il6 and Nfat in mice did not alter their ethanol preference. These findings suggest that interactions between Lrrk2 gene expression, the immune system and behavior influence the abusive consumption of ethanol. The better understanding of the factors associated with the neurobiology of ethanol addiction may help to develop new therapeutic targets for this disease. The study of Yang et al. investigated the association between dietary factors and asthma using Mendelian randomization and the IEU Open GWAS project as the source of exposure and outcome datasets, considering various dietary factors (e.g., alcohol intake, processed meat intake, fruit intake, etc.). The results indicated that alcohol intake frequency was associated with an increased risk of asthma, while fresh fruit intake and dried fruit intake were found to be protective factors against asthma. However, no significant causal relationship was observed between asthma and the other dietary factors studied. These findings contribute to our understanding of the impact of specific dietary components on asthma risk and emphasize the importance of considering dietary factors in respiratory health research.

Collectively, the articles in this Research Topic showed important aspects of the role played by dietary habits in the microbiota-immune system axis and in the immune-mediated disease pathogenesis. Furthermore, the researchers pointed out for the importance to consider the nutrition and microbiota modulation as important factors that can be used as a preventive and therapeutic tool in immune-mediated diseases.

## Author contributions

MP, JV, and GO wrote the editorial. All authors approved the final version.
